# Visual Tracking of Hydrogen Sulfide: Application of a Novel Lysosome-Targeted Fluorescent Probe for Bioimaging and Food Safety Assessment

**DOI:** 10.3390/molecules29163906

**Published:** 2024-08-18

**Authors:** Likun Liu, Yitong Liu, Haoqing Ren, Peng Hou, Haijun Wang, Jingwen Sun, Lei Liu, Chuan He, Song Chen

**Affiliations:** 1 Research Institute of Medicine & Pharmacy, Qiqihar Medical University, Qiqihar 161006, China; 2 College of Pharmacy, Qiqihar Medical University, Qiqihar, 161006, China13313575607@139.com (H.R.); houpeng1982@163.com (P.H.);

**Keywords:** hydrogen sulfide, fluorescence probe, turn-on response, bioimaging, foodstuff

## Abstract

The equilibrium state of hydrogen sulfide (H_2_S), a gaseous signaling molecule produced by lysosomal metabolites, in vivo is crucial for cellular function. Abnormal fluctuations in H_2_S concentration can interfere with the normal function of lysosomes, which has been closely linked to the pathogenesis of a variety of diseases. In view of this, a novel fluorescent probe **Lyso-DPP** based on 1,3,5-triarylpyrazolines was developed for the precise detection of H_2_S in lysosomes by using the hydrophilic morpholine moiety as a lysosomal targeting unit, and 2,4-dinitroanisole as a fluorescence-quenching and H_2_S-responsive unit. The probe cleverly combines the advantages of simple synthesis, sensitive blue fluorescence turn-on with a limit of detection, LOD, of 97.3 nM, good stability, and fast response time (10 min), which makes **Lyso-DPP** successful in portable monitoring of meat freshness in the form of test strips. Moreover, the excellent biocompatibility and precise targeting capability of the probe **Lyso-DPP** make it perform well in the monitoring of H_2_S in lysosomes, living cells, and zebrafish. This work not only provides new technical tools for food quality control but also paves up new ideas for early diagnosis and treatment of H_2_S-related diseases.

## 1. Introduction

Lysosomes are unique organelles in eukaryotic cells, known for their acidic environment (pH maintained between 4.5 and 5.5) and their abundance of hydrolytic enzymes. These enzymes not only serve as sites for intracellular waste disposal and degradation of biomolecules but also participate in a wide range of physiological processes, such as cellular autophagy, apoptosis, pathogen clearance, and cell signaling [1,2,3,4]. The activity of hydrolytic enzymes within lysosomes is essential for the stability of the intracellular environment and the maintenance of cellular functions [5]. Abnormalities in lysosomal function can interfere with critical physiological processes in organisms, leading to pathologies such as lysosomal storage diseases, renal diseases, inflammatory responses, and cancer [6,7].

During lysosomal metabolism, sulfur-containing amino acids, such as L-cysteine, generate hydrogen sulfide (H_2_S) through specific enzymatic reactions, for instance, by cystathionine-β-synthasecysteine (CBS), 3-mercaptopyruvate sulfotransferase (3-MST), and cystathionine-γ-lyase (CSE) [8,9]. H_2_S, as an endogenous gaseous signaling molecule, participates in the regulation of cellular signaling and has a critical role in the homeostasis of a wide range of physiological and pathological processes. Maintaining homeostasis is crucial for human health [10,11,12]. However, abnormal fluctuations in hydrogen sulfide concentration can cause lysosomal dysfunction and are also associated with pathological activity in Parkinson’s disease, diabetes mellitus, Alzheimer’s disease, gastric mucosal damage, and certain types of cancer, and these altered pathologies may have a profound impact on an individual’s longevity and quality of life [13,14]. In addition, hydrogen sulfide, a toxic gas with a typical rotten egg odor, is released in elevated concentrations during food spoilage when bacteria decompose sulfides in food, a phenomenon that can serve as an important indicator of freshness in protein-rich meat products [15,16]. In view of the important role of hydrogen sulfide in food quality and human health, it is particularly urgent to develop accurate assays to assess the levels of hydrogen sulfide in food and biological samples and even lysosomes. This will help improve food safety standards, enhance our awareness of the biological functions of hydrogen sulfide, and contribute to the scientific basis for the prevention and treatment of related diseases.

Fluorescence sensing technology has become an indispensable analytical tool in biomedical research due to its unique advantages. It not merely causes minimal damage to organisms but enables precise spatial localization and temporal tracking at the molecular level, demonstrating excellent spatial and temporal resolution [17,18,19,20]. With the continuous progress of science and technology, fluorescence sensing technology has developed a variety of fluorophores, such as naphthalimide, coumarin, phenothiazine, BODIPY, etc. These fluorophores have different chemical properties and biocompatibility, which can meet the needs of different biological detection applications [21,22,23,24]. Among these fluorophores, pyrazoline derivatives stand out for their excellent blue fluorescence emission, low cytotoxicity, high fluorescence quantum yield, and good photostability, which make them a highlight in the field of fluorescence sensing [25,26,27]. Currently, many fluorescent probes for H_2_S detection in various complex environments and biological samples have been reported based on the aforementioned fluorophores utilizing reaction mechanisms like nitroxide reduction, azide reduction, nucleophilic substitution, and sulfurolysis (Table S1). Nevertheless, the available fluorescent probes are still insufficient in the face of the constant challenges of disease pathology research, especially for the specific detection of H_2_S in complex organelles such as lysosomes. As an important intracellular site of digestion and degradation, changes in H_2_S levels in lysosomes are closely associated with the development of a variety of diseases [28]. Therefore, the development of fluorescent probes that can specifically target lysosomes and monitor the dynamic changes of H_2_S in lysosomes in real-time is of great significance for revealing the specific mechanisms of H_2_S in the pathological processes of diseases and exploring new therapeutic targets for diseases.

Based on the above, in order to overcome the limitations of the existing technology in the detection of H_2_S in lysosomes, the lysosome-targeted fluorescent probe **Lyso-DPP** was synthesized by using pyrazolines modified with a hydrophilic morpholine moiety as a fluorophore skeleton in combination with 2,4-dinitroanisole as a response moiety, and the probe itself exhibits a very low fluorescence background due to the fluorescence-quenching effect of dinitroanisole. After triggering the nucleophilic substitution reaction of H_2_S, the rapid departure of the quencher 2,4-dinitroanisole resulted in a remarkable blue fluorescence response of the probe **Lyso-DPP** in aqueous solution, which was accompanied by a rapid turn-on of fluorescence (<10 min), a low limit of detection (97.3 nM), and superior specificity. Based on the favorable photophysical properties, we realized the detection of H_2_S in food spoilage by loading the probe into a portable paper-based assay. In bioimaging, **Lyso-DPP** successfully achieved outstanding visualization of exogenous H_2_S in HepG2 cells and endogenous H_2_S in HeLa cells and effectively monitored H_2_S at the zebrafish in vivo level. In addition, the fluorescence response of **Lyso-DPP** to H_2_S in a weakly acidic environment shows favorable adaptability, which enables the probe to adapt to the lysosomal environment and localize lysosomes more precisely, thus providing a powerful tool to precisely analyze the specific mechanism of H_2_S in cell metabolism, signaling, and even pathological processes.

## 2. Results and Discussion

### 2.1. Design and Synthesis of ***Lyso-DPP***

**Scheme 1:**
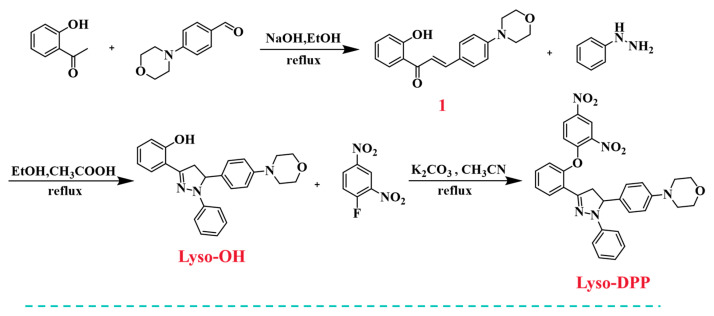
Synthesis process of **Lyso-DPP**.

In this study, we employed a multistep synthetic strategy to successfully construct a novel fluorescent probe **Lyso-DPP** (Scheme 1). First, we selected analytically pure 4-(4-morpholinyl)benzaldehyde and *O*-hydroxyacetophenone as starting materials and introduced a lysosome-targeted morpholino moiety into the molecular structure via an efficient condensation reaction. Further, **Lyso-OH**, a novel fluorophore based on the 1,3,5-triarylpyrazoline backbone, was synthesized using the intramolecular ring-forming reaction of phenylhydrazine with compound **1**. 2,4-dinitrophenyl ether was carefully selected as the quencher of the fluorophore and the functional group specifically recognizing H_2_S in the final stage of the synthesis of the probe **Lyso-DPP**. This design strategy ensures that the probe exhibits a low fluorescence state when it is not reacted with H_2_S, whereas once affected by H_2_S, the dissociation of 2,4-dinitrophenyl ether leads to the release of the fluorophore **Lyso-OH**, which enables the sensitive and selective detection of H_2_S. We performed an exhaustive structural characterization of the intermediate 1, the fluorophore **Lyso-OH**, and the final probe **Lyso-DPP** generated during the synthesis by nuclear magnetic resonance (NMR) spectroscopy and high-resolution mass spectrometry (HRMS) techniques (Figures S1–S9).

### 2.2. Photophysical Properties of ***Lyso-DPP***

Following the successful synthesis of the fluorescent probe **Lyso-DPP**, we first probed the photophysical properties of **Lyso-DPP** using UV-Vis absorption and fluorescence emission spectra in EtOH-PBS buffer (*v*/*v* = 3/7, pH = 7.4, 20.0 mM) to evaluate its potential for fluorescence detection of H_2_S. As shown in Figure S10, we recorded the UV-Vis absorption spectra of **Lyso-OH** (10.0 μM) and **Lyso-DPP** (10.0 μM) in the presence or absence of H_2_S (100.0 μM). It was observed that the probe **Lyso-DPP**, which did not react with H_2_S, exhibited its maximum absorption peak at 372 nm. In contrast, when the probe **Lyso-DPP** reacted with H_2_S, its absorption peak underwent a slight blue shift to 359 nm, a change that coincided with the absorption peak of **Lyso-OH**. This blue shift of the absorption peak explains the mechanism of fluorescence enhancement of the probe **Lyso-DPP** upon reaction with H_2_S from a spectroscopic point of view. Namely, the dissociation of 2,4-dinitrophenyl leads to the exposure of the fluorophore **Lyso-OH**, which restores its fluorescence properties.

**Figure 1:**
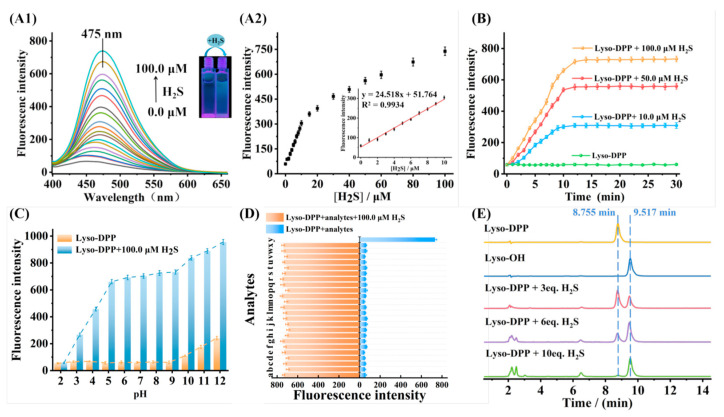
(**A1**) Fluorescence spectra of **Lyso-DPP** (10.0 μM) after reaction with H_2_S (0.0–100.0 μM) in EtOH-PBS buffer (*v*/*v* = 3/7, pH = 7.4, 20.0 mM), inset: colorimetric image of **Lyso-DPP** before and after reaction with H_2_S under hand-held UV light; and changes in the corresponding fluorescence intensity at 475 nm (**A2**), inset: a linear relationship between the fluorescence intensity of **Lyso-DPP** (10.0 μM) and H_2_S (0.0–10.0 μM). (**B**) Plot of fluorescence response of **Lyso-DPP** with H_2_S versus time. (**C**) Fluorescence response of **Lyso-DPP** in the presence or absence of H_2_S at pH 2–12. (**D**) Fluorescence responses of **Lyso-DPP** (10.0 μM) to potential interferents (100.0 μM) and anti-interference test of **Lyso-DPP** (10.0 μM) to H_2_S (a-y: Na^+^, Mg^2+^, Ca^2+^, Zn^2+^, NH_4_^+^, Arg, Cys, Asn, Ile, Met, Ser, Val, Tyr, Glu, Phe, Lys, Hcy, NO_3_^−^, ONOO^−^, SO_3_^2−^, CO_3_^2−^, ClO_4_^−^, H_2_O_2_, HCIO, H_2_S) at pH = 7.4. (**E**) HPLC chromatogram of probe **Lyso-DPP** (yellow), **Lyso-OH** (blue), and reaction mixtures of **Lyso-DPP** with 3.0 eq H_2_S (red)/6.0eq H_2_S (purple)/10.0eq H_2_S (green). Conditions: eluent, CH_3_CN/H_2_O (*v*/*v*, 80/20), flow rate 3.0 mL/min, temperature 25 °C, injection volume 10.0 μL. λ_ex_ = 360 nm and λ_em_ = 475 nm; slit: 5.0 nm/10.0 nm.

In further studies, we delved into the fluorescence response properties of the fluorescent probe **Lyso-DPP** to H_2_S. Through the analysis of fluorescence emission spectra (Figure 1A1,A2), the fluorescence signal of the probe **Lyso-DPP** was extremely weak when it did not react with H_2_S. However, with the gradual increase of H_2_S concentration, the fluorescence intensity of the probe at 475 nm exhibited an obvious concentration-dependent enhancement, which was increased 13-fold with the addition of 100.0 μM H_2_S. An excellent linear response relationship of **Lyso-DPP** to low concentrations of H_2_S in the range of 0.0–10.0 μM was found by quantitative analysis (y = 24.518x + 51.764, R^2^ = 0.9934). The limit of detection (LOD) of **Lyso-DPP** for H_2_S was calculated to be 97.3 nM according to Equation 3δ/k (k represents the slope of the linear relationship exhibited between the **Lyso-DPP** fluorescence intensity and the change in hydrogen sulfide (H_2_S) concentration at a specific wavelength of 475 nm. δ is the standard deviation of the fluorescence intensity measurements of the blank sample. Calculated by repeating the measurement of the fluorescence spectrum of a blank sample of **Lyso-DPP** 10 times, we were able to assess the repeatability of the experiment and the level of background noise [29]). The above results indicate that the probe **Lyso-DPP** has high sensitivity and good potential for quantitative analysis in H_2_S detection.

### 2.3. Kinetic Study of ***Lyso-DPP***

The fast response capability of small-molecule fluorescence sensors is crucial for capturing dynamic processes of bio-molecules during real-time monitoring and in situ imaging. To fully evaluate the performance of the **Lyso-DPP** fluorescent sensor, we conducted an in-depth study of its temporal kinetic properties (Figure 1B). In the control experiment without H_2_S, the fluorescence intensity of the blank probe solution did not change significantly during the observation period of 30 min, which indicates that **Lyso-DPP** has excellent chemical stability in aqueous solution. When different concentrations of H_2_S (10.0–100.0 μM) were introduced, **Lyso-DPP** exhibited significant fluorescence enhancement, and the fluorescence intensity was gradually enhanced with the prolongation of incubation time. In particular, **Lyso-DPP** achieved rapid fluorescence turn-on for all tested concentrations of H_2_S within 10 min and maintained its maximum fluorescence for the following 20 min. The rapid change in fluorescence of the test system reflects that **Lyso-DPP** can track dynamic changes in H_2_S concentration in real time.

### 2.4. pH Study of ***Lyso-DPP***

In biological systems, different levels of microenvironments each maintain specific pH conditions. For this reason, we further investigated the ability of **Lyso-DPP** to detect H_2_S in a wide pH range of 2–12 (Figure 1C). The fluorescence signal of the blank probe set remained stable and quenched in aqueous solutions at pH 2–9. When the pH was 2–5, the detection ability of the probes for H_2_S was gradually enhanced until the fluorescence signal reached the maximum fluorescence intensity at pH = 5, and the fluorescence intensity remained stable in the pH range of 5–9. Furthermore, a significant increase in fluorescence intensity was observed when the pH was increased to 10–12, which might be due to the decomposition of the probe structure under alkaline conditions. The above results indicate that **Lyso-DPP** can achieve a precise response to H_2_S in a pH environment of 5–9.

### 2.5. Selectivity and Competition Study of ***Lyso-DPP***

In order to deeply explore the specificity of the probe **Lyso-DPP** for the detection of H_2_S in complex biological systems, the response of **Lyso-DPP** to a variety of potential interferents was investigated, including cations (Na^+^, Mg^2+^, Ca^2+^, Zn^2+^, NH_4_^+^), anions (NO_3_^−^, ClO_4_^−^, SO_3_^2−^, CO_3_^2−^), amino acids (Arg, Cys, Asn, Ile, Met, Ser, Val, Tyr, Glu, Phe, Lys, Hcy), and reactive oxygen species (ONOO^−^, H_2_O_2_, HCIO) [30,31,32]. A significant enhancement of the fluorescence signal was not observed when the probe **Lyso-DPP** was exposed to a blank solution or contacted with the above-interfering substances. On the contrary, once **Lyso-DPP** was in contact with H_2_S, its fluorescence intensity at 475 nm increased significantly, a phenomenon visualized in Figure 1D. In addition, even in a complex environment where H_2_S coexists with other interferences, **Lyso-DPP** was still able to exhibit a pronounced blue fluorescence response. These results not only confirmed the high specificity of **Lyso-DPP** in detecting H_2_S but also demonstrated its potential application under complex physiological conditions.

### 2.6. Sensing Mechanism

**Scheme 2:**
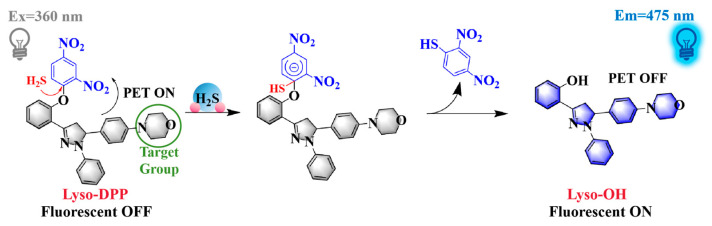
The proposed response pattern of **Lyso-DPP** to H_2_S.

Based on the spectral analysis and in-depth study of the existing literature [33,34], we hypothesized the response mechanism of the probe **Lyso-DPP** to H_2_S (Scheme 2) as follows: due to the Photoinduced Electron Transfer (PET) process, **Lyso-DPP** has almost no fluorescence signal when it does not react with H_2_S. However, when **Lyso-DPP** came into contact with H_2_S, the nucleophilic nature of H_2_S led to a rapid nucleophilic substitution reaction between it and **Lyso-DPP**, which led to the breakage of the dinitroanisole bond and was accompanied by the release of the fluorophore **Lyso-OH**. In order to verify this reaction mechanism, the high-performance liquid chromatography (HPLC) technique was employed to analyze the probe **Lyso-DPP**, the fluorophore **Lyso-OH**, and the components of the reaction mixture in detail (Figure 1F). The results showed that **Lyso-DPP** and **Lyso-OH** in the free state presented characteristic peaks on the chromatogram at 8.755 and 9.517 min, respectively. When the **Lyso-DPP** was mixed with H_2_S, we noticed that the characteristic peaks of the probe molecules originally located at 8.755 min gradually decreased with the increase of H_2_S concentration. In comparison, the peaks of the fluorophores located at 9.517 min increased accordingly. When an excess of H_2_S (10 equivalents) was added, only the characteristic peak at 9.517 min was observed in the reaction mixture. This further confirmed the completeness of the nucleophilic substitution reaction and the complete release of the fluorophore. Furthermore, to further verify the identity of the reaction product, high-resolution mass spectrometry (HRMS) analysis of the main peak detected at *m*/*z* = 400.2017 was consistent with the expected mass of the fluorophore **Lyso-OH** (Figure S11), thus providing conclusive evidence for the reaction mechanism.

### 2.7. Fluorescence Imaging in Live Cells

Given the excellent performance demonstrated by probe **Lyso-DPP** in aqueous solution, we were motivated to further explore its potential for imaging H_2_S in a cellular environment. Prior to the study of its imaging capabilities, cytotoxicity was first evaluated on HepG2 hepatocytes and HeLa cervical cancer cells. MTT colorimetry was used to assess the potential effects of the probe on these cell lines (Figure S12). The experimental results revealed that the survival rate of both cell types remained above 80% even at probe concentrations as high as 40.0 μM, suggesting that **Lyso-DPP** has low cytotoxicity, which provides an important prerequisite for the application of the probe in live cell imaging.

**Figure 2:**
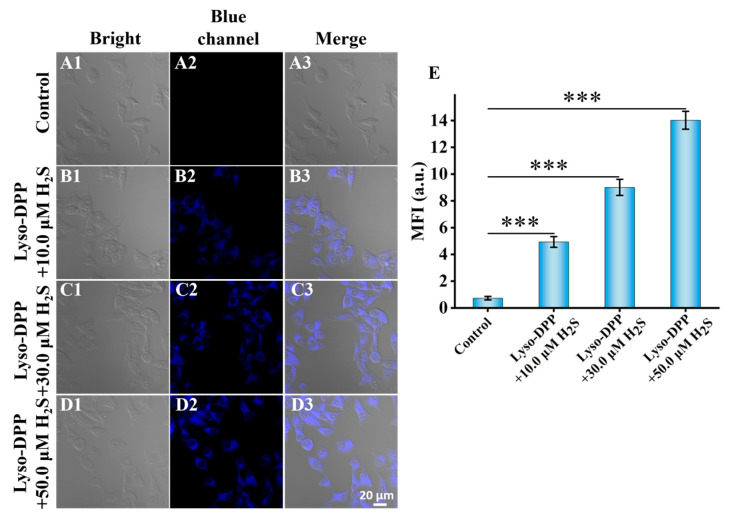
Confocal imaging of HepG2 cells. (**A1**–**A3**) HepG2 cells were only treated with 10.0 μM **Lyso-DPP** for 30 min. (**B1**–**B3**/**C1**–**C3**/**D1**–**D3**) HepG2 cells were pretreated with H_2_S (10.0/30.0/50.0 μM) for 30 min, followed by the addition of 10.0 μM **Lyso-DPP** for another 30 min. (**E**) Fluorescence intensities in panel (**A1**–**D3**). *n* = 3, error bars were ±SD. Statistical analysis was performed using one-way ANOVA. Among them, *** *p* < 0.001. Blue channel: λ_ex_ = 405 nm, λ_em_ = 425–475 nm.

Subsequently, the ability of **Lyso-DPP** to detect exogenous H_2_S in HepG2 cells was assessed by incubating HepG2 cells with different concentrations of H_2_S (10.0, 30.0, and 50.0 μM) for 30 min. The cells were imaged after being washed three times with sterilized PBS and introduced to **Lyso-DPP** (10.0 μM) to continue the incubation for 30 min. Figure 2 showed that the fluorescence signal in the blue channel was enhanced with the increase of H_2_S concentration, indicating that **Lyso-DPP** was able to effectively detect cellular exogenous H_2_S.

**Figure 3:**
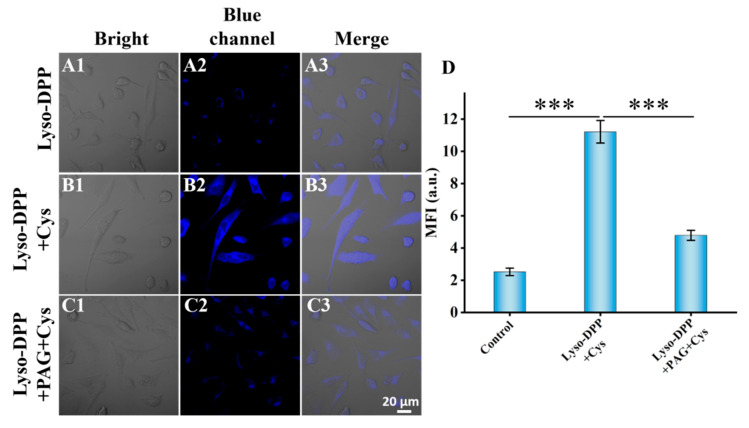
Confocal imaging of HeLa cells. (**A1**–**A3**) HeLa cells were only treated with 10.0 μM **Lyso-DPP** for 30 min. (**B1**–**B3**) HeLa cells were pretreated with Cys (200.0 μM) for 1 h, followed by the addition of 10.0 μM **Lyso-DPP** for another 30 min. (**C1**–**C3**) HeLa cells were sequentially treated with PAG (1.0 mM) for 30 min and Cys (200.0 μM) for 1 h, followed by the addition of 10.0 μM **Lyso-DPP** for 30 min. (**D**) Fluorescence intensities in panel A-C. *n* = 3, error bars were ±SD. Statistical analysis was performed using one-way ANOVA. Among them, *** *p* < 0.001. Blue channel: λ_ex_ = 405 nm, λ_em_ = 425–475 nm.

Next, HeLa cells were selected to explore the ability of **Lyso-DPP** to detect cellular endogenous H_2_S (Figure 3). Since cysteine (Cys) can be converted to H_2_S intracellularly by the enzymatic reaction of cystathionine-γ-lyase (CSE), 200.0 μM Cys was chosen to incubate the cells for 1 h, and then **Lyso-DPP** (10.0 μM) was co-incubated with the cells for 30 min. A significantly enhanced fluorescence signal was observed when compared to the control group that was incubated with the probe **Lyso-DPP** only, indicating that Cys treatment significantly increased the level of endogenous H_2_S in the cells. To further demonstrate that the strong fluorescence signal generated by the Cys-incubated cells was due to the production of endogenous H_2_S, the same treatment described above was performed on the cells after selecting 1.0 mM propargylglycine (PAG, a common CSE inhibitor) for preincubation of the cells for 30 min, and a substantial attenuation of fluorescence intensity was observed. It was shown that Cys was able to increase the elevation of endogenous H_2_S levels as well as monitor the levels of endogenous and exogenous H_2_S by **Lyso-DPP** in the cells.

**Figure 4:**
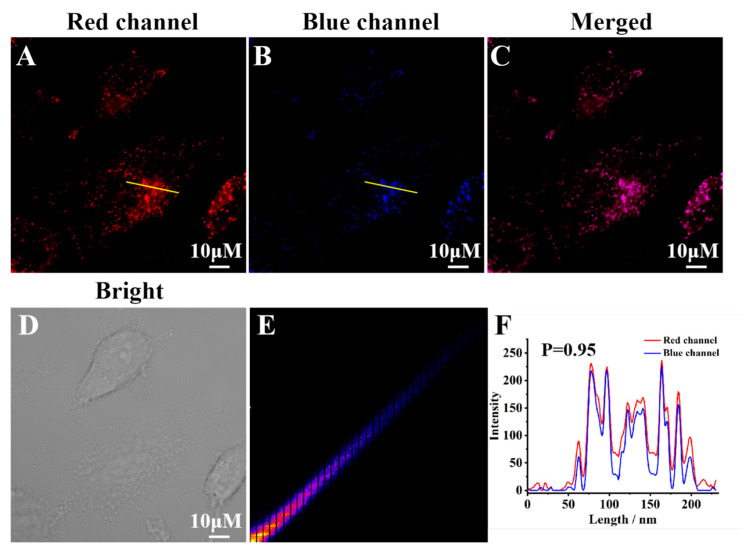
Co-localization imaging of **Lyso-DPP** and Lyso Tracker Red in HeLa cells. (**A**) Fluorescence imaging of Lyso Tracker Red at 580–620 nm; (**B**) Fluorescence imaging of **Lyso-DPP** at 425–475 nm. (**C**) Merged fluorescence image. (**D**) Bright image. (**E**) Correlation analysis of fluorescence intensity between (**A**) and (**B**). (**F**) Fluorescence intensity distribution of the red channel (Lyso Tracker Red) and the blue channel (Lyso-DPP) on the yellow line.

In order to deeply investigate the specific localization ability of **Lyso-DPP** for H_2_S in lysosomes, a commercial lysosome-targeting dye (Lyso Tracker Red, 1.0 μM) was used as a control, and HeLa cells were co-incubated with **Lyso-DPP** (10.0 μM) for 30 min (Figure 4). A significant overlap in the spatial distribution of the red fluorescent signal from Lyso Tracker Red and the blue fluorescent signal from **Lyso-DPP** was observed by fluorescence confocal microscopy imaging. This overlap suggests that the **Lyso-DPP** probe can specifically localize to lysosomes. The co-localization coefficient (Pearson’s coefficient), which quantifies the extent of this co-localization, had a high value of 0.95, further confirming the specific localization of **Lyso-DPP** to lysosomes.

### 2.8. Fluorescence Imaging in Zebrafish

**Figure 5:**
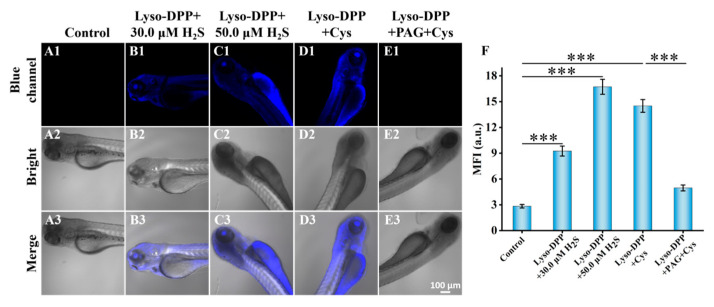
Confocal imaging of zebrafish. (**A1**–**A3**) Zebrafish were only treated with 10.0 μM **Lyso-DPP** for 30 min. (**B1**–**B3**/**C1**–**C3**) Zebrafish were pretreated with H_2_S (30.0/50.0 μM) for 30 min, followed by the addition of 10.0 μM **Lyso-DPP** for another 30 min. (**D1**–**D3**) Zebrafish were pretreated with Cys (200.0 μM) for 1 h, followed by the addition of 10.0 μM **Lyso-DPP** for another 30 min. (**E1**–**E3**) Zebrafish were sequentially treated with PAG (1.0 mM) for 30 min and Cys (200.0 μM) for 1 h, followed by the addition of 10.0 μM **Lyso-DPP** for 30 min. (**F**) Fluorescence intensities in panel (**A1**–**E3**). *n* = 3, error bars were ± SD. Statistical analysis was performed using one-way ANOVA. Among them, *** *p* < 0.001. Blue channel: λ_ex_ = 405 nm, λ_em_ = 425–475 nm.

In view of the high transparency of zebrafish embryos and the high homology between their genes and humans [35,36], 3-day-old zebrafish embryos were used as model organisms in this study to evaluate the bioimaging performance of **Lyso-DPP** and its sensitivity to the detection of H_2_S under in vivo conditions by fluorescence imaging. The experimental results exhibited (Figure 5) that the blue fluorescence intensity of zebrafish embryos was gradually enhanced with increasing H_2_S concentration (from 10.0 μM to 50.0 μM), which was similar to the previous observation in HepG2 cells. This increase in fluorescence intensity compared to the control group incubated with only 10.0 μM of **Lyso-DPP** probe indicates that **Lyso-DPP** can effectively detect exogenous H_2_S in zebrafish embryos.

Further, to explore the ability of **Lyso-DPP** to detect endogenous H_2_S, cysteine was employed as an inducer of endogenous H_2_S, and PAG was used to inhibit the conversion process of Cys to H_2_S. The experimental results indicated that 200.0 μM Cys treatment significantly increased the fluorescence intensity of zebrafish embryos, in contrast to the untreated control. Meanwhile, after PAG pretreatment, the fluorescence intensity of zebrafish embryos was significantly reduced even in the presence of Cys, suggesting that the enhancement of the fluorescence signal is indeed associated with the production of endogenous H_2_S. This finding further confirms the ability of **Lyso-DPP** to specifically detect endogenous and exogenous H_2_S in living zebrafish embryos.

### 2.9. Detection of H_2_S and Food Samples by Means of Test Strips

The results of the spectral analysis confirmed that the probe **Lyso-DPP** is capable of quantitatively detecting H_2_S in the blue fluorescence channel. To validate the potential application of the probe in real-world environments, we further developed **Lyso-DPP** test strips for the detection of different concentrations of H_2_S (0.0–100.0 μM). The changes in fluorescence intensity of the test strips were recorded under irradiation by a 365 nm UV lamp (Figure S13). The experimental results showed that the blue fluorescence intensity of the test strips increased accordingly with the increase in H_2_S concentration, verifying that the **Lyso-DPP** test strips had a sensitive response to H_2_S.

**Figure 6:**
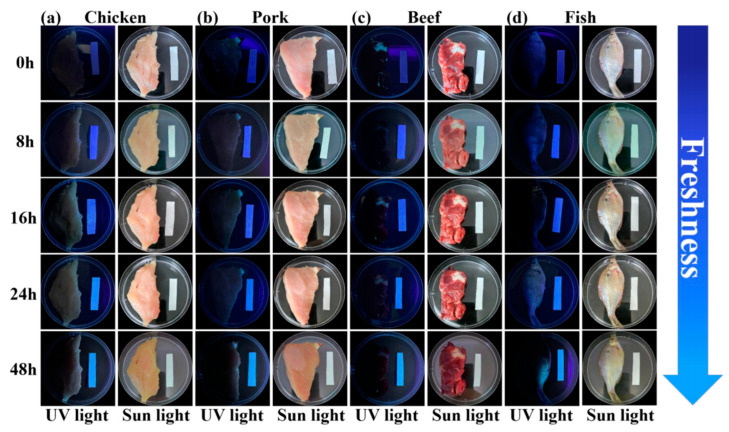
Detection of H_2_S by **Lyso-DPP** test strips during spoilage of (**a**) chicken, (**b**) pork, (**c**) beef, and (**d**) fish.

Considering that the degradation of sulfides by microorganisms releases H_2_S during the deterioration process of meat, we further explored the application of **Lyso-DPP** test strips in meat freshness testing (Figure 6). For the experiment, we selected chicken, pork, beef, and fish samples of the same quality and placed them in Petri dishes containing **Lyso-DPP** test strips. At a constant temperature of 30 °C, we monitored the changes in the fluorescence intensity of the test strips. At the initial moment (0 h), the **Lyso-DPP** test strips did not display a fluorescent response, indicating that the food samples were in a fresh state. With time, a gradual increase in fluorescence intensity was observed, and this change was proportional to the amount of H_2_S released, thus reflecting a decrease in the freshness of the meat. This simple and rapid assay not only demonstrated the potential application of the **Lyso-DPP** probe in H_2_S detection but also provided a practical tool for food safety monitoring.

## 3. Materials and Methods

### 3.1. Reagents and Apparatus

The solvents and reagents used in the experiments were purchased from commercial suppliers and were of analytically pure grade, which could be directly applied to the experimental process without additional purification steps. Meat and seafood samples were selected from the local market. Nuclear magnetic resonance (NMR) spectra of the compounds were acquired by Bruker Advance 600 MHz spectrometer. Ultraviolet-visible (UV-Vis) spectra and fluorescence spectral data were obtained by Unico UV-4802 spectrophotometer and Shimadzu RF-6000 fluorescence spectrophotometer. The bioimaging section was captured using a Zeiss (Oberkochen, Germany) LSM710 Wetzlar laser scanning confocal microscope.

### 3.2. Synthesis of ***Lyso-OH***

4-(4-morpholinyl)benzaldehyde (344.1 mg, 1.8 mmol), *O*-hydroxyacetophenone (245.1 mg, 1.8 mmol), and NaOH (144.0 mg, 3.6 mmol) were dissolved in anhydrous ethanol (12 mL). The reaction mixture was refluxed at 80 °C for 4 h. After cooling, the solid precipitated out. The crude product was then washed with ice-cold ethanol and vacuum filtered to yield the orange-red solid compound **1**. (342.0 mg, 61.3%). ^1^H NMR (600 MHz, *d_6_*-DMSO) δ 12.98 (s, 1H), 8.28 (dd, J = 8.0, 1.4 Hz, 1H), 7.89–7.78 (m, 4H), 7.58–7.52 (m, 1H), 7.05–6.95 (m, 4H), 3.74 (t, J = 6.0 Hz, 4H), 3.28 (t, J = 6.0 Hz, 4H). ^13^C NMR (151 MHz, *d_6_*-DMSO) δ 193.21, 162.04, 152.77, 145.69, 135.84, 130.41, 124.16, 120.30, 118.80, 117.54, 116.39, 113.80, 65.69, 46.78. HRMS (*m*/*z*): calcd for [M + H]^+^ 310.1443; found, 310.1429.

Compound **1** (618.8 mg, 2.0 mmol) and phenylhydrazine (2.0 mL, 22.0 mmol) were dissolved in 10 mL of anhydrous ethanol, and the mixture was refluxed at 80 °C for 3 h. After that, glacial acetic acid (7.2 mL, 126.0 mmol) was added to the reaction system, and the reaction continued for 2 h. After the reaction was complete, the solution was cooled to precipitate solid, and ethanol was used for recrystallization to obtain a light yellow solid **Lyso-OH** (280.0 mg, 35.1%). ^1^H NMR (600 MHz, *d_6_*-DMSO) δ 10.59 (s, 1H), 7.41 (d, J = 7.70 Hz, 1H), 7.28 (t, J = 7.70 Hz, 1H), 7.19 (dd, J = 15.1, 7.80 Hz, 4H), 6.99 (d, J = 8.20 Hz, 1H), 6.97–6.88 (m, 5H), 6.76 (t, J = 7.30 Hz, 1H), 5.37 (dd, J = 12.00, 6.60 Hz, 1H), 3.99 (dd, J = 17.60, 12.10 Hz, 1H), 3.70 (t, J = 6.0 Hz, 4H), 3.23 (dd, J = 17.60, 6.60 Hz, 1H), 3.06 (t, J = 6.0 Hz, 4H). ^13^C NMR (151 MHz, *d_6_*-DMSO) δ 156.69, 150.52, 144.17, 130.82, 129.53, 128.52, 127.20, 120.03, 119.60, 117.11, 116.52, 115.88, 113.45, 66.54, 62.23, 48.69, 44.26. HRMS (*m*/*z*): calcd for [M + H]^+^ 400.2025; found, 400.2016.

### 3.3. Synthesis of ***Lyso-DPP***

**Lyso-OH** (399.5 mg, 1.0 mmol), 2,4-dinitrofluorobenzene (0.2 mL, 1.6 mmol), and potassium carbonate (110.6 mg, 0.8 mmol) were placed into 15 mL of acetonitrile and refluxed at 90 °C for 5 h. At the end of the reaction, 50 mL of water was added, extracted by methylene chloride, and washed by saturated saline. The organic layer was dried over anhydrous sodium sulfate and concentrated to obtain the crude product, which was purified by silica gel column chromatography (petroleum ether:ethyl acetate = 2:1) to yield the orange solid, probe **Lyso-DPP** (379.8 mg, 67.2%). ^1^H NMR (600 MHz, *d_6_*-DMSO) δ 8.93 (d, J = 2.7 Hz, 1H), 8.41 (dd, J = 9.3, 2.8 Hz, 1H), 7.93–7.75 (m, 1H), 7.64–7.44 (m, 2H), 7.37 (d, J = 7.8 Hz, 1H), 7.01 (t, J = 7.9 Hz, 2H), 6.94 (dd, J = 14.0, 9.0 Hz, 3H), 6.77 (d, J = 8.7 Hz, 2H), 6.65 (t, J = 8.9 Hz, 3H), 5.31 (dd, J = 12.1, 5.6 Hz, 1H), 3.86 (dd, J = 17.3, 12.2 Hz, 1H), 3.68 (t, J = 6.0 Hz, 4H), 3.05 (dd, J = 17.4, 5.6 Hz, 1H), 3.01 (t, J = 6.0 Hz, 4H). ^13^C NMR (151 MHz, *d_6_*-DMSO) δ 156.28, 149.54, 143.61, 138.67, 132.57, 130.22, 129.09, 127.83, 126.89, 125.97, 123.35, 122.45, 119.29, 118.01, 115.64, 113.29, 66.51, 62.31, 48.61, 44.78. HRMS (*m*/*z*): calcd for [M + H]^+^ 566.2040; found, 566.2014.

### 3.4. Spectral Measurement

**Lyso-DPP** was prepared by dissolving it in DMSO to form a probe stock solution at a concentration of 0.3 mM, which was further dissolved in a mixed ethanol/PBS solution (*v*/*v* = 3/7, pH = 7.4) to obtain the final probe solution. 1.0 mM Na_2_S was solubilized in ultrapure water to form a stabilized donor solution of H_2_S. This donor solution was then diluted to a series of different concentration gradients, depending on the experimental requirements, for fluorescence spectroscopy at an excitation wavelength of 360 nm, with an excitation slit width of 5 nm and an emission slit width of 10 nm. Other interferences (Na^+^, Mg^2+^, Ca^2+^, Zn^2+^, NH_4_^+^, Arg, Cys, Asn, Ile, Met, Ser, Val, Tyr, Glu, Phe, Lys, Hcy, NO_3_^−^, ONOO^−^, SO_3_^2−^, CO_3_^2−^, ClO_4_^−^, H_2_O_2_, HCIO) were prepared as stock solutions in pure water at a concentration of 3.0 mM for selection and competition experiments.

### 3.5. Cell Culture and Incubation

HepG2 cells and HeLa cells were cultured in DEME medium supplemented with 10% fetal bovine serum at 37 °C with 5% CO_2_. Cells were inoculated into 96-well plates and incubated for 24 h. Cells were adhered to the wall and then incubated with different concentrations of probe solution (0.0, 10.0, 20.0, 30.0, 40.0 μM) for 24 h. Cells were washed three times with sterilized PBS and then added with MTT solution (5 mL/mg) and incubated for 4 h. Cell activity was analyzed quantitatively by measuring the absorbance at 490 nm.

In this study, HepG2 cells were selected to assess the ability to detect exogenous H_2_S. In control experiments, cells were incubated with a 10.0 μM fluorescent probe for 30 min only. Comparatively, in the experimental group, cells were first pre-treated with 10.0–50.0 μM H_2_S solution for 30 min to simulate the exposure environment of exogenous H_2_S, followed by incubation with the same concentration of probe for 30 min to observe the fluorescence response of the probe to H_2_S.

For the detection of endogenous H_2_S, HeLa cells were selected for imaging experiments. The incubation conditions of the control group were identical to those of the control group for exogenous H_2_S. In the experimental group, we used two different treatments. First, cells were pretreated with 200.0 μM Cys for 1 h to promote endogenous H_2_S production and then incubated with a fluorescent probe for imaging. Second, another set of HeLa cells was preloaded with propargylglycine (PAG, 1.0 mM, an inhibitor of CSE enzyme) for 30 min, after which they continued to receive treatments of Cys (200.0 μM) and **Lyso-DPP** (10.0 μM) for 1 h and 30 min, respectively. Prior to fluorescence imaging, all cell samples were washed three times with sterile phosphate buffer solution.

### 3.6. Zebrafish Incubation

Three-day-old zebrafish provided by Nanjing EzeRinka Biotechnology Co., Ltd (Nanjing, China) and used for in vivo imaging of H_2_S. Zebrafish were first incubated with only 10.0 μM probe solution for 30 min as a control. For the determination of exogenous H_2_S, zebrafish were stained with 10.0 μM probe solution for 30 min based on incubation with different concentrations of H_2_S (30.0 μM, 50.0 μM), whereas for endogenous H_2_S, zebrafish were divided into two groups, one group of zebrafish was treated with 200.0 μM Cys for 1 h, and the other group was treated with 200.0 μM Cys in zebrafish loaded with 1.0 mM PAG for 30 min, followed by incubation of both groups with 10.0 μM probe solution. The incubation was followed by three washes with sterile PBS before imaging. All animal-related experimental manipulations strictly followed the rigorous review and formal approval by the Animal Ethics Committee of Qiqihar Medical University (approval number QMU-AECC-2024-111).

### 3.7. Test Strips Test Processing

To explore the application of the **Lyso-DPP** probe in the detection of H_2_S, filter papers were first cut into rectangular shapes of 1 cm width and 3 cm length. Next, these paper strips were immersed in **Lyso-DPP** solution at a concentration of 50.0 μM for 20 min to fully adsorb the probe molecules. Subsequently, the paper strips were removed and placed in air to dry to ensure that the probe molecules were uniformly immobilized on the filter paper. Following this, two different sets of experimental treatments were performed. First, the dried filter paper was incubated with different concentrations of H_2_S (0.0–100.0 μM) for 20 min to simulate the fluorescence response at different H_2_S concentrations in order to construct a fluorescence colorimetric card of H_2_S for quantitatively analyzing the concentration of H_2_S in the actual environment. Next, the same filter paper was directly exposed to fresh meat and seafood samples to verify the probe’s ability to detect food freshness. The fluorescence changes of the filter paper were observed under a 365 nm handheld UV lamp.

## 4. Conclusions

In summary, an H_2_S “turn-on” fluorescent probe **Lyso-DPP** based on pyrazoline derivatives was designed, and the probe itself appears fluorescence-quenched due to the PET effect. When contacted with H_2_S, the probe undergoes a rapid nucleophilic substitution reaction, leading to the appearance of strong blue fluorescence at 475 nm. This fluorescence enhancement not only provides an intuitive and sensitive signal for the detection of H_2_S but also greatly improves the convenience and sensitivity of H_2_S monitoring during meat spoilage through the application of test paper format. In addition, thanks to the low cytotoxicity and high specificity for H_2_S, **Lyso-DPP** is able to accurately reflect the concentration changes of H_2_S in living cells as well as zebrafish, demonstrating excellent imaging results. Notably, the clever incorporation of the morpholine moiety in **Lyso-DPP** endowed it with precise lysosomal targeting ability, which enabled the probe to show extraordinary advantages in the detection of H_2_S in lysosomes. The present study not only provides a new tool for real-time monitoring of H_2_S but also offers a new perspective for a deeper understanding of the role of H_2_S in cellular physiology and pathological processes through precise lysosomal targeting.

## Data Availability

The data presented in this study are available in the Supplementary Materials.

## Supplementary Materials

The following supporting information can be downloaded at https://www.mdpi.com/article/10.3390/molecules29163906/s1, Table S1. Comparison of the proposed probe with other reported fluorescence probes for the detection of H_2_S. Figure S1. ^1^H NMR spectrum of compound **1** in DMSO-*d6*. Figure S2. ^13^C NMR spectrum of compound **1** in DMSO-*d6*. Figure S3. HRMS spectrum of compound **1**. Figure S4. ^1^H NMR spectrum of **Lyso-OH** in DMSO-*d6*. Figure S5. ^13^C NMR spectrum of **Lyso-OH** in DMSO-*d6*. Figure S6. HRMS spectrum of **Lyso-OH**. Figure S7. ^1^H NMR spectrum of probe **Lyso-DPP** in DMSO-*d6*. Figure S8. ^13^C NMR spectrum of probe **Lyso-DPP** in DMSO-*d6*. Figure S9. HRMS spectrum of probe **Lyso-DPP**. Figure S10. The absorption spectra of probe **Lyso-DPP** (10.0 μM), **Lyso-OH** (10.0 μM) and the test solution added with H_2_S (100.0 μM). Figure S11. HRMS spectrum of **Lyso-DPP** + H_2_S. Figure S12. Cytotoxicity assays of probe **Lyso-DPP** at different concentrations (0.0–40.0 μΜ) for HepG2 cells and HeLa cells. Figure S13. The probe **Lyso-DPP** (10.0 μM) responds to fluorescence color changes of test paper from 0.0–100.0 μM H_2_S under 365 nm UV light irradiation. Figure S14. Fluorescence responses at 475nm of **Lyso-DPP** (10.0 μM) to potential interferents (100.0 μM) and anti-interference test of **Lyso-DPP** (10.0 μM) to H_2_S (a-y: Na^+^, Mg^2+^, Ca^2+^, Zn^2+^, NH_4_^+^, Arg, Cys, Asn, Ile, Met, Ser, Val, Tyr, Glu, Phe, Lys, Hcy, NO_3_^−^, ONOO^−^, SO_3_^2−^, CO_3_^2−^, ClO_4_^−^, H_2_O_2_, HCIO, H_2_S) at pH=5.5. Figure S15. Fluorescence intensity changes at 475nm of probe **Lyso-DPP** (10.0 μM) after giving a treatment with GSH (1.0 mM) and H_2_S (100.0 μM).
