# Supplementary material for: Visual Tracking of Hydrogen Sulfide: Application of a Novel Lysosome-Targeted Fluorescent Probe for Bioimaging and Food Safety Assessment
Source: Molecules. 2024 Aug 18;29(16):3906. doi: 10.3390/molecules29163906 (PMC11357609; doi:10.3390/molecules29163906)
Supplement: Supplementary file 1 [file molecules-29-03906-s001.zip › molecules-3166611-supplementary.pdf]

Supporting information

Visual tracking of hydrogen sulfide: application of a novel lysosome-targeted fluorescent probe for bioimaging and food safety assessment

Likun Liu[a], Yitong Liu[b], Haoqing Ren[b], Peng Hou[b], Haijun Wang[b], Jingwen Sun[b], Lei Liu[b], Chuan He[b], Song Chen[b,*]

[a] Research Institute of Medicine & Pharmacy, Qiqihar Medical University, Qiqihar, 161006, China

[b] College of Pharmacy, Qiqihar Medical University, Qiqihar, 161006, China

[*]Corresponding author,

E-mail address: chensongchemistry@163.com

Table of contents

Page

Table S1..**S1**

Figure S1-3..**S3**

Figure S4-6..**S4**

Figure S7-9..**S5**

Figure S10-12..**S6**

Figure S13-14..**S7**

Figure S15..**S8**

Table S1. Comparison of the proposed probe with other reported fluorescence probes for the detection of H_2S.

Probe	Organelle targeting	LOD	Response time	Test system (pH=7.4)	Applications	Literature
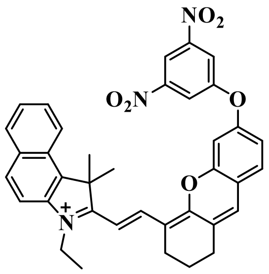	NO	23.0 nM	30 min	PBS buffer	A549 cell<br>Mice	Sensors and Actuators: B. Chemical<br>339<br>(2021)<br>129881
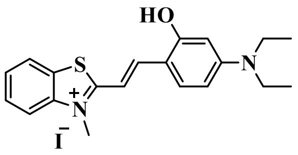	Mitochondria	99.7 nM	30 s	DMSO/H_2O<br>(5/5, v/v)	HeLa cell<br>Water samples<br>Beer samples	Journal of Photochemistry & Photobiology, A: Chemistry<br>444<br>(2023)<br>114993
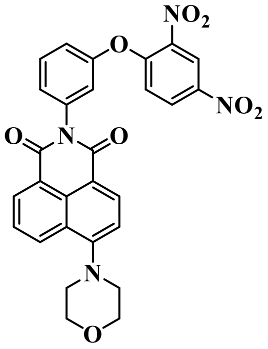	Lysosome	9.8 nM	15 min	DMSO/H_2O<br>(1/99, v/v)	A549 cell<br>Mouse slide	Spectrochimica Acta Part A: Molecular and Biomolecular Spectroscopy<br>249<br>(2021)<br>119311
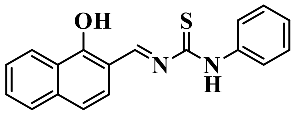	NO	0.82 µM	30 min	PBS	HeLa cell	Journal of Photochemistry & Photobiology, A: Chemistry<br>456<br>(2024)<br>115829
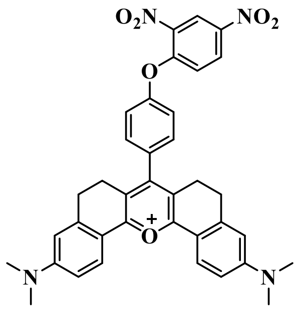	NO	0.48 µM	30 min	DMSO/PBS<br>(1/9, v/v)	Environmental water samples<br>Food samples<br>HeLa cell	Journal of Food Composition and Analysis<br>128<br>(2024)<br>106022

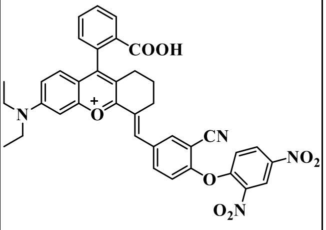	NO	0.52 µM	40 min	DMSO/PBS (2/8, v/v)	A549 cell<br>Rice root	Spectrochimica Acta Part A: Molecular and Biomolecular Spectroscopy 321 (2024) 124762
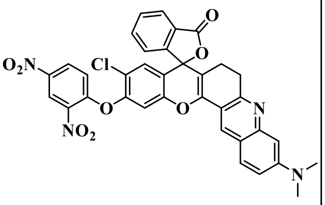	NO	0.33 µM	15 min	DMSO/PBS (1/1, v/v)	Food samples<br>LLC cell	Spectrochimica Acta Part A: Molecular and Biomolecular Spectroscopy 315 (2024) 124250
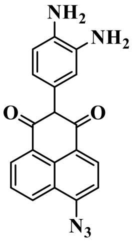	Lysosome	1.13 µM	120 s	DMSO/PBS (6/4, v/v)	Various cells<br>Wild-type C. elegans<br>Zebrafish	Dyes and Pigments 228 (2024) 112224
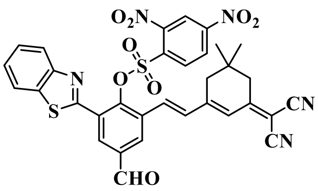	Mitochondria	31.5 µM	60 s	HEPES	HeLa cell<br>Mice	Journal of Molecular Structure 1286 (2023) 135576
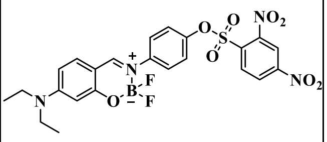	NO	76.0 nM	20 min	PBS	HeLa cell	Journal of Fluorescence 33 (2023) 1603-1608
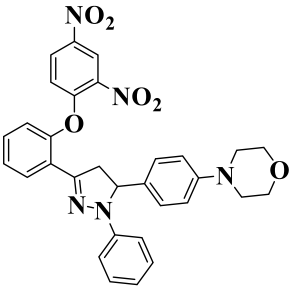	**Lysosome**	**97.3 nM**	**10 min**	**C_2H_5OH/PBS (3/7,v/v)**	**Food samples<br>HeLa cell<br>Zebrafish**	**This work**

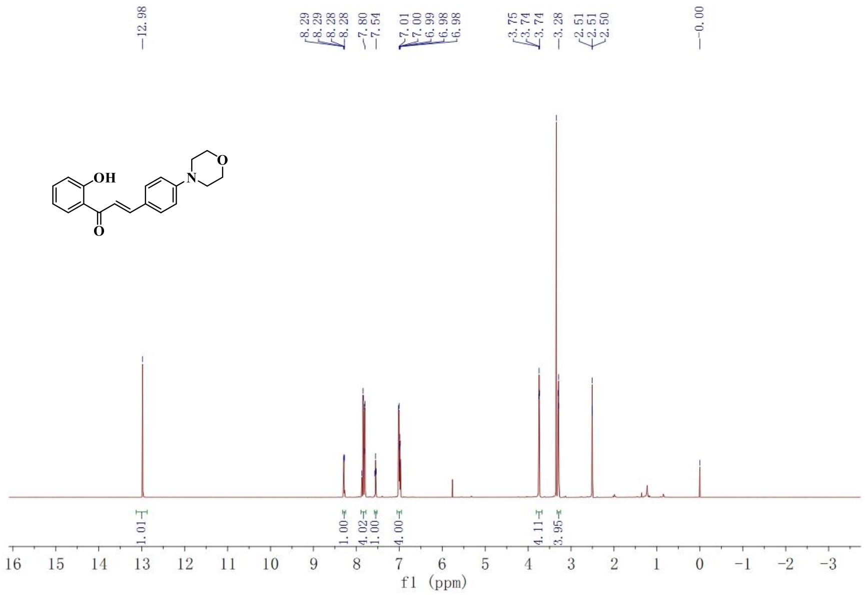

Figure. S1. ^{1}H NMR spectrum of compound **1** in DMSO-d_6.

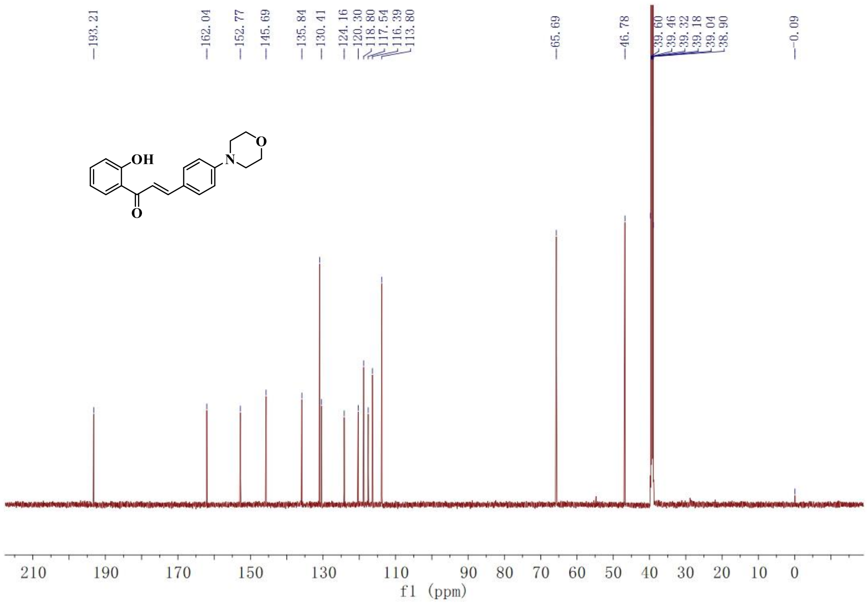

Figure. S2. ^{13}C NMR spectrum of compound **1** in DMSO-d_6.

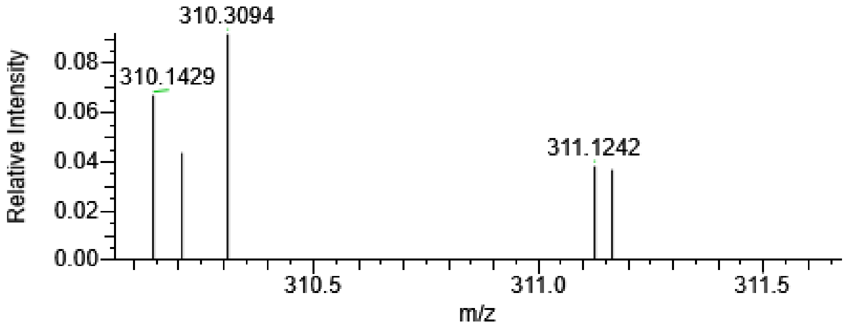

Figure. S3. HRMS spectrum of compound **1**.

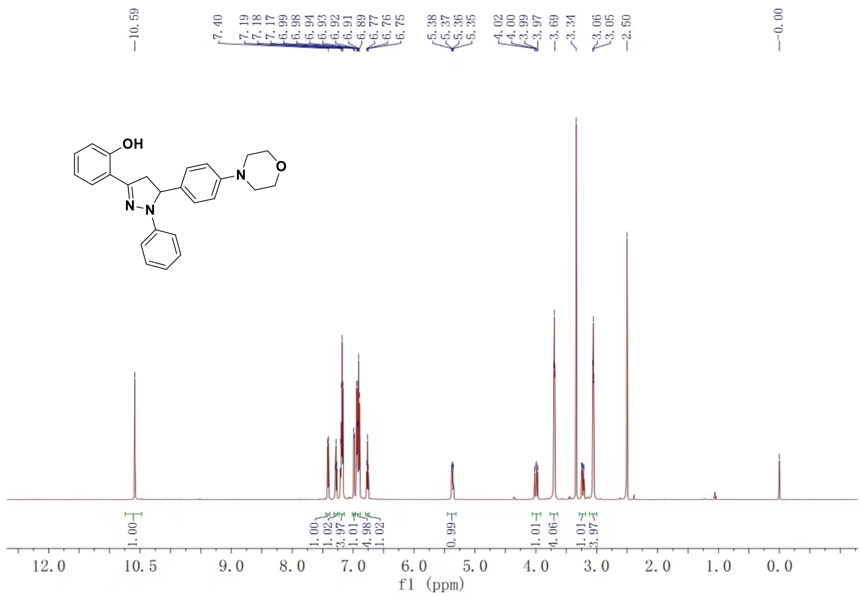

Figure. S4. ^{1}H NMR spectrum of **Lyso-OH** in DMSO-d_6.

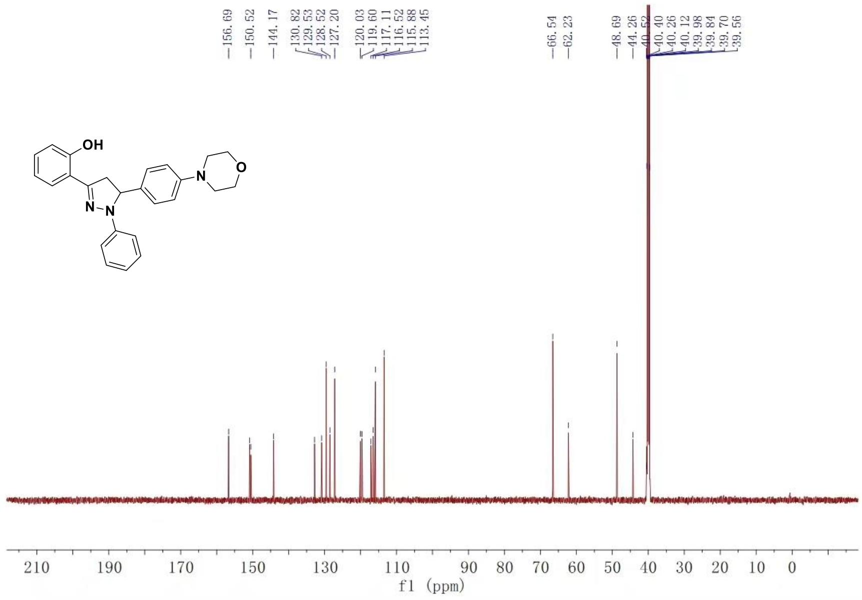

Figure. S5. ^{13}C NMR spectrum of **Lyso-OH** in DMSO-d_6.

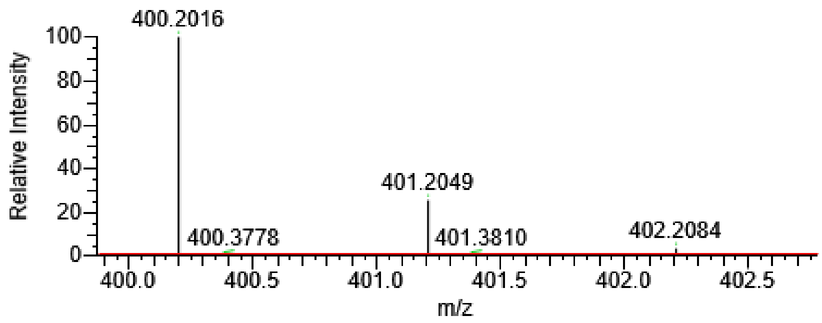

Figure. S6. HRMS spectrum of **Lyso-OH**.

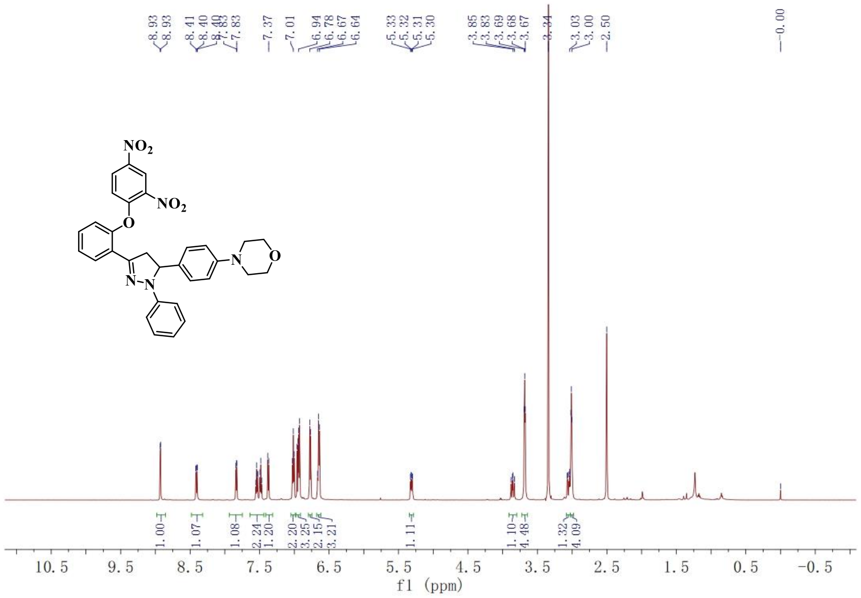

Figure. S7. ^{1}H NMR spectrum of probe **Lyso-DPP** in DMSO-d_6.

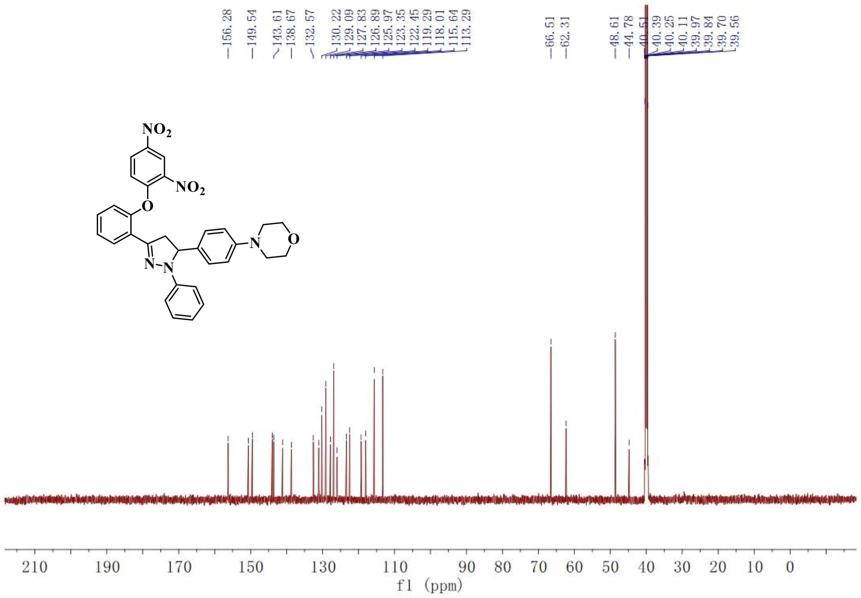

Figure. S8. ^{13}C NMR spectrum of probe **Lyso-DPP** in DMSO-d_6.

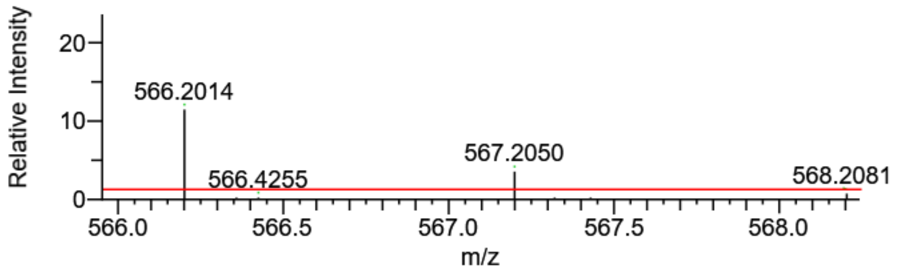

Figure. S9. HRMS spectrum of probe **Lyso-DPP**.

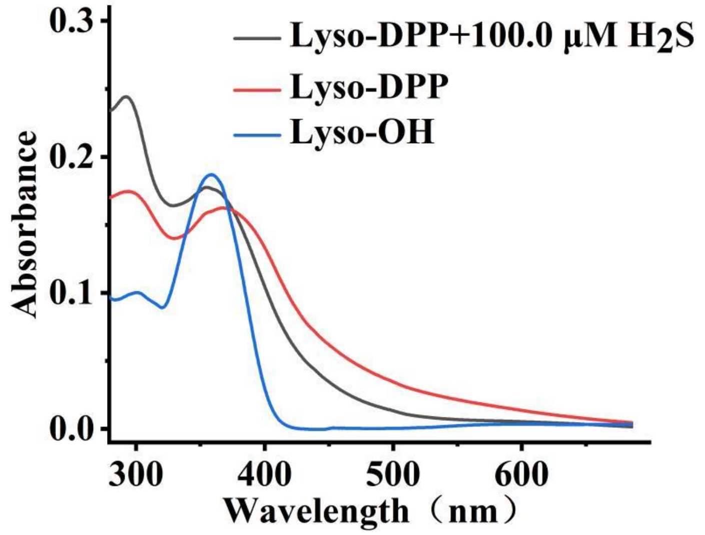

Figure. S10. The absorption spectra of probe **Lyso-DPP** (10.0 μM), **Lyso-OH** (10.0 μM) and the test solution added with H_2S(100.0 μM).

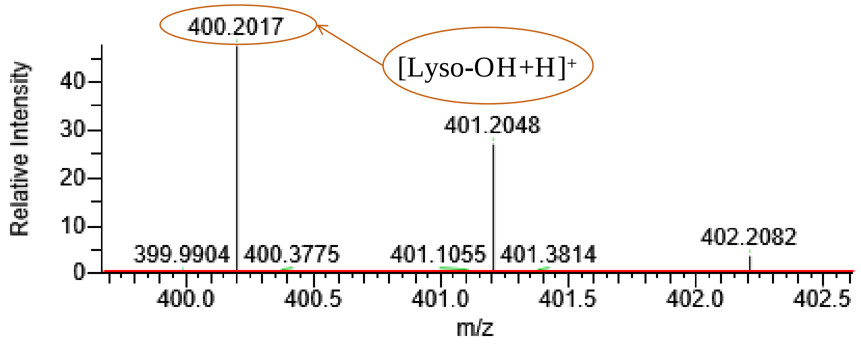

Figure. S11. HRMS spectrum of **Lyso-DPP** + H_2S.

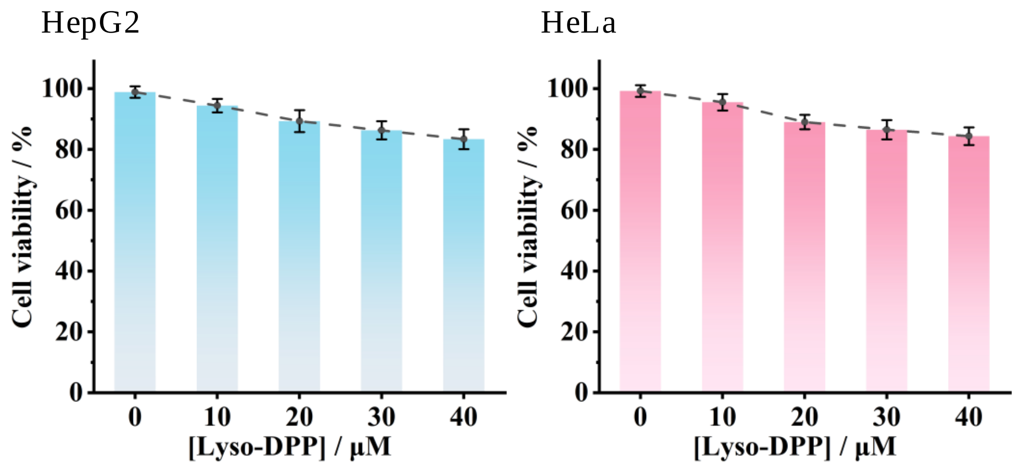

Figure. S12. Cytotoxicity assays of probe **Lyso-DPP** at different concentrations (0.0-40.0 μM) for HepG2 cells and HeLa cells.

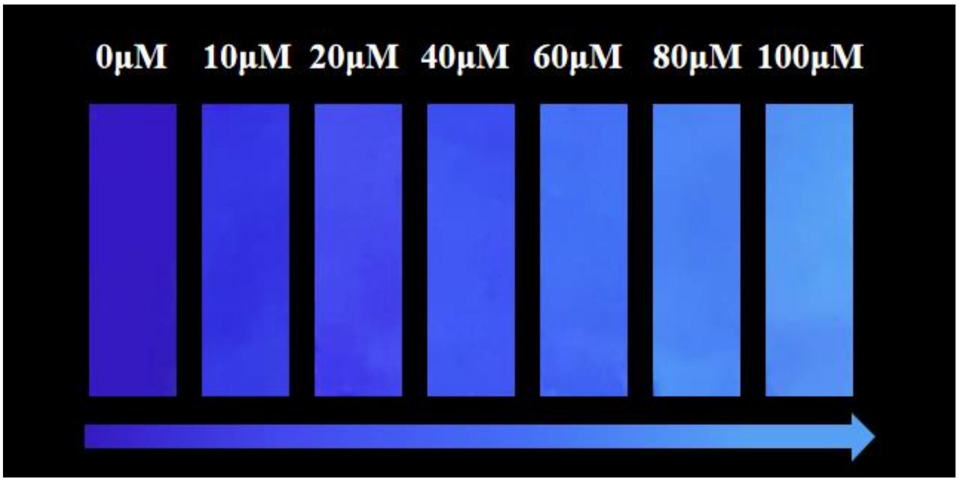

Figure. S13. The probe **Lyso-DPP** (10.0 μM) responds to fluorescence color changes of test paper from 0.0-100.0 μM H_2S under 365 nm UV light irradiation.

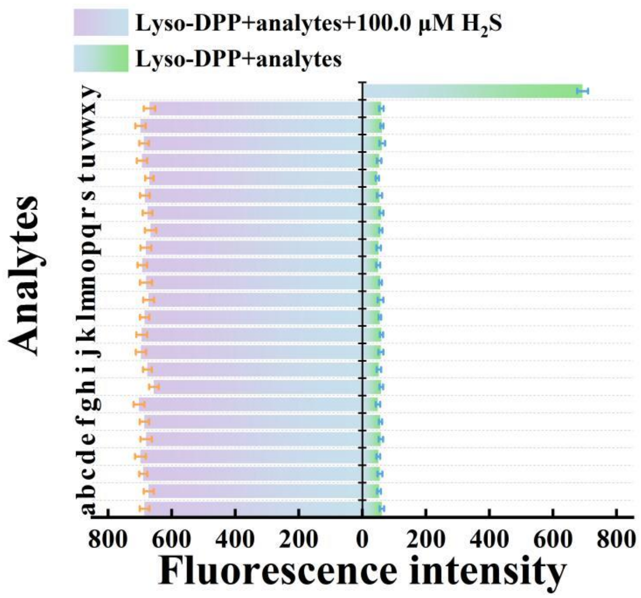

Figure. S14. Fluorescence responses at 475nm of **Lyso-DPP** (10.0 μM) to potential interferents (100.0 μM) and anti-interference test of **Lyso-DPP** (10.0 μM) to H_2S (a-y: Na^+, Mg^{2+}, Ca^{2+}, Zn^{2+}, NH_4^+, Arg, Cys, Asn, Ile, Met, Ser, Val, Tyr, Glu, Phe, Lys, Hcy, NO_3^-, $ONOO^-$, SO_3^{2-}, CO_3^{2-}, ClO_4^-, H_2O_2, HClO, H_2S) at pH=5.5.

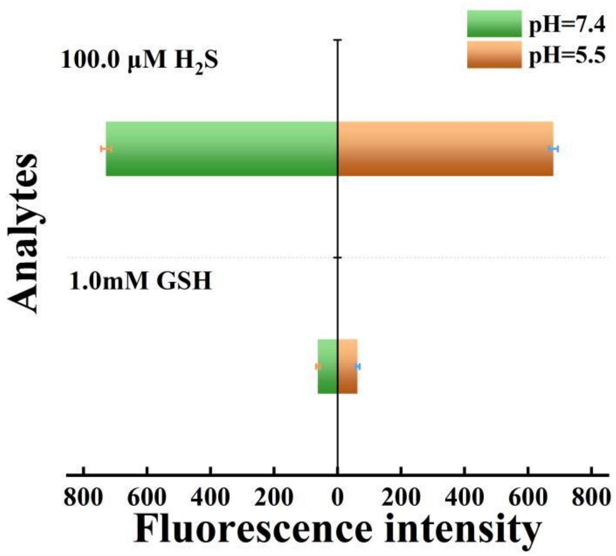

Figure. S15. Fluorescence intensity changes at 475nm of probe **Lyso-DPP** (10.0 μM) after giving a treatment with GSH (1.0 mM) and H_2S (100.0 μM).
